# Prevalence and Determinants of Peripheral Neuropathy Among Adult Type II Diabetes Mellitus Patients Attending a Non-communicable Disease Clinic in Rural South India

**DOI:** 10.7759/cureus.15493

**Published:** 2021-06-07

**Authors:** Prakash Mathiyalagen, Sivagami Kanagasabapathy, Zubaidabegum Kadar, Anandaraj Rajagopal, Kavita Vasudevan

**Affiliations:** 1 Epidemiology and Public Health, Indira Gandhi Medical College and Research Institute, Puducherry, IND

**Keywords:** diabetes mellitus, peripheral neuropathy, michigan neuropathy screening instrument, smoking, duration of diabetes, hyperglycemia

## Abstract

Background

Diabetic peripheral neuropathy (DPN) is one of the most common microvascular complications of diabetes. Almost half of the diabetic patients develop foot ulcer as a complication of DPN during their lifetime. The aim was to estimate the prevalence and identify the risk factors of diabetic peripheral neuropathy among adult diabetes mellitus (DM) patients.

Methods

A cross-sectional study was conducted among 421 type 2 DM patients attending Non-Communicable Disease (NCD) clinic in rural Puducherry through systematic random sampling. The study instruments used for data collection were a pre-tested semi-structured questionnaire, Michigan Neuropathy Screening Instrument (MNSI), Morisky Green Levine Scale (MGLS), physical measurements and recent laboratory results. The data was captured using Epicollect5 and analyzed using SPSS version 20.

Results

The prevalence of DPN was 31.1% (95% confidence interval (CI): 27.1%-35.1%). The mean age, duration of diabetes, and duration of foot symptoms were 57.91±10.61, 7.00±6.23, 5.56±5.26 years. Smoking (adjusted odds ratio (AOR) 3.14; 95% CI 1.73-5.69), mean duration of diabetes>5years (AOR 2.74; 95% CI 1.71-4.40), hyperglycemic status(>200mg/dl) (AOR 2.24; 95% CI 1.08-4.64) and unemployment (AOR 2.05; 95% CI 1.11-3.76) were found to be statistically significant determinants of DPN on binary logistic regression analysis.

Conclusions

A considerable proportion of diabetics are at risk of developing DPN among rural DM patients. More diligent screening in a primary health care setting and addressing the modifiable risk factors like smoking, obesity, physical inactivity, and uncontrolled hyperglycemia will delay or hamper DPN development among diabetic patients.

## Introduction

Diabetes is one of the non-communicable diseases, the prevalence of which is increasing world-wide. Type 1 diabetes is due to lack of insulin, whereas type 2 is due to insulin resistance. As per World Health Organization (WHO) global report, the number of adults living with diabetes is 422 million. The prevalence of diabetes in South-East Asian Region (SEAR) is 96 million [1]. In India, men with high blood sugar levels are 8.8% and 7.4% in urban and rural regions. In comparison, women with high blood sugar levels are 6.9% and 5.2% in urban and rural regions, respectively. In Puducherry, the prevalence of women with high blood sugar levels is 7.3%, and men with high blood sugar levels are 7.5% [2]. Diabetes and its complications have a significant impact not only on the individuals' economy but also on their quality of life [3]. As the disease progresses, it can involve multiple organs, and the complications can be broadly divided into macrovascular and microvascular. One of the microvascular complications is diabetic peripheral neuropathy. Proper management of diabetes and screening of complications will have a potential impact on the quality of life of these patients.

Diabetic peripheral neuropathy (DPN) is the most common cause of neuropathy world-wide. The Toronto Consensus meeting defined typical DPN as asymmetrical and length-dependent sensorimotor polyneuropathy attributed to metabolic and micro-vessel alterations as a result of long-standing hyperglycaemia and metabolic derangements [4]. DPN has caused severe challenges to health expenditures globally. Effectively controlling the substantial health expenditures resulting from DPN is a world-wide concern. Neuropathy increases the risk of foot ulcers, infection and the eventual need for limb amputation. The rates of amputation are higher among people with diabetes [5]. The chronic peripheral neuropathy associated with diabetes represents an insidious process, and the pathological severity is poorly linked with the development of symptoms. As the incidence of type 2 Diabetes Mellitus increases every year, it has been recommended that prevention or early diagnosis of DPN should occur at the primary care level. There are many tools for screening DPN like Diabetic neuropathy symptom score, Neuropathy disability score, Neuropathy symptom score, Toronto clinical scoring system and Michigan Neuropathy Screening Instrument (MNSI). MNSI has been used in this study which has a sensitivity of 80% and specificity of 95% [6]. MNSI has got a kappa value of 0.588 [7]. MNSI consists of two parts: the first part is a questionnaire about symptoms of DPN and the second part consists of foot examination [8].

This study has been done at the primary health care level, the first contact point for rural diabetic patients. If screening of DPN can be done at the primary health care level, it would be of great benefit for diabetic patients. When peripheral neuropathy is detected earlier, it can curb further complications like foot ulcers and amputations and improve the quality of life among diabetic patients. This study aimed to determine the proportion of individuals with DPN and the determinants associated with DPN development among diabetic patients attending the rural health training centre in Puducherry.

## Materials and methods

Study design, period and setting

A facility-based descriptive cross-sectional study was done from February 1 to April 30, 2020, at the Non-Communicable Disease (NCD) clinic of Community Health Centre (CHC), Karikalampakkam Puducherry, India. Karikalampakkam is Indira Gandhi Medical College and Research Institute and is located 13km away from the college. The catchment area of this rural health centre roughly covers around 35,000 population. NCD clinic is being conducted every Tuesday and Friday, where around 80 to 100 adult DM patients are being treated in a single NCD clinic day. This rural centre was purposefully chosen due to feasibility, the largest rural health centre in Puducherry, and many enrolled Diabetes Mellitus (DM) patients in the NCD clinic.

Selection criteria

Inclusion Criteria

All individuals who attended the NCD clinic, aged more than 30 years and diagnosed as having type II DM for a minimum of one-year duration, were considered study participants.

Exclusion Criteria

Those patients with stroke and below-knee amputation were excluded from the study.

Sample size calculation and sampling technique

Considering the prevalence of DPN among rural DM patients as 52.9% [5], alpha error as 5%, absolute precision as 5% and substituting these values in the single proportion formula, the minimum sample size was 383. With a non-response rate of 10%, it was decided to collect data from a minimum of 421 study participants. Study participants were chosen based on systematic random sampling by considering every second eligible participant until the desired sample size was achieved.

Study tool

A pre-tested semi-structured questionnaire was used to collect data regarding their socio-demographic characteristics and other variables like physical activity, duration of diabetes, duration of foot symptoms, recent fasting blood sugar (FBS), Post-prandial blood sugar (PPBS), Random blood sugar (RBS), mode of a treatment since the diagnosis, compliance to medications (based on Morisky Green Levine Scale), height, weight, waist circumference, hip circumference and foot examination (based on Michigan Neuropathy Screening Instrument part 2).

Ethical consideration

Institute Ethics Committee (Indira Gandhi Medical College and Research Institute, Puducherry) approval was obtained before beginning the study (No.6/sl.No.247/27th-IEC -2020). Informed written consent was taken from the study participants after explaining the pros and cons of the study procedure by the National Ethical Guidelines for Biomedical and Health Research involving Human Participants. The data collectors have trained adequately before beginning the study.

Operational definitions

Michigan Neuropathy Screening Instrument Scoring (MNSI)

The second part of MNSI is a brief physical assessment involving 1) inspection of the feet for deformities, dry skin, hair or nail abnormalities, callous or infection 2) semi-quantitative assessment of vibration sensation at the dorsum of the great toe 3) Grading of ankle reflex and 4) monofilament testing (The touch sensation was tested in ten different points using 10g monofilament). On a ten-point scale, a score of greater than two is considered as having peripheral neuropathy [8].

Cut-Off Value for Diagnosis of Diabetes

Diabetes is diagnosed either when fasting blood sugar is more than or equal to 126mg/dl, post-prandial blood sugar is more than or equal to 200mg/dl, or random blood sugar is more than or equal to 200 mg/dl according to the American Diabetes Association guidelines.

Body Mass Index (BMI)

BMI was calculated using the standard formula (weight in kilograms/ height in meters squared), and patients were classified according to WHO classification [9].

Morisky Green Levine Scale (MGLS)

Compliance with medication was measured using four items MGLS questionnaire [10]. If the answer to anyone question is yes, then it is considered non-compliant for medication.

Foot Symptoms Free Duration

It was calculated as age at the onset of diabetes minus age at the onset of foot symptoms.

Mode of Treatment

The modality of treatment adopted within six months of diagnosis of DM. It can be either insulin, insulin along with oral hypoglycemic agent or oral hypoglycemic agent alone.

Literate

A person aged seven years and above, who can read and write in any language with understanding, is considered literate as per Census 2011. 

Alcohol Consumption

A current alcoholic is a person who has consumed alcohol in the past 30 days. Past alcoholic refers to the person who has consumed alcohol in the past but not in the past 30 days. Never alcoholic refers to the person who has not consumed alcohol in his or her lifetime.

Other Measurements and Testing

Measurements like height, weight, hip circumference and waist circumference were taken following standard procedures [11], and waist-hip ratio (WHR) was calculated. Ankle reflex, the great toe vibration sensation, and monofilament testing were done per standard guidelines [12].

Statistical Methods

Data was captured using Epicollect5 and analyzed using Statistical Package for the Social Sciences (SPSS) version 20. The normality of the data was assessed using the Shapiro-Wilk test. Categorical data were expressed as frequencies and percentages and continuous data as mean and standard deviation or median and interquartile range based on the normality of the data. Chi-square test, Students t-test and Mann-Whitney U test were used to determine the association between a dependent variable and independent variables. Binary logistic regression was used to determine the confounding variables and adjusted odds ratio (AOR). A p-value less than 0.05 was considered for statistical significance.

## Results

Clinical and demographic characteristics of study participants

A total of 421 diabetic patients participated in the study. Among the study participants, 62.9% were females, 48.7% were literates, 34% were employed, 80.5% were married, 20.9% were smokers, and 23% were alcoholics. Around 45% of the participants indulged themselves in moderate-intensity physical activity like walking. All the study participants were invariably prescribed multi-vitamins. The mean age, duration of diabetes, BMI, and WHR participants were 57.91 ±10.60 years, 7±6.23 years, 25.96 kg/m2 and 0.96, respectively.

Prevalence of diabetic peripheral neuropathy

The proportion of study participants with DPN based on MNSI score was 31.1% (95% confidence interval (CI) 27.1%-35.1%). Among them, 18.8% were females, and 12.4% were males.

Determinants of diabetic peripheral neuropathy

Table 1 shows the various risk factors associated with DPN. Age, educational status, occupation, smoking, physical activity, RBS, BMI, and duration of diabetes were the statistically significant risk factors associated with DPN in the univariate analysis.

**Table 1:** Qualitative factors associated with the risk of diabetic peripheral neuropathy (n=421) *Chi-square test, DM: Diabetes mellitus, WHR: Waist hip ratio, BMI: Body mass index, DPN: Diabetic peripheral neuropathy, OR: Odds ratio, CI: Confidence interval, MNSI: Michigan Neuropathy Screening Instrument

Variables	DPN Present (MNSI >2) n (%)	DPN Absent (MNSI < / =2) n (%)	p-value*	OR (95% CI)
Age≥60 years	60 (45.8)	88 (30.3)	0.003	1.94 (1.26-2.96)
Female	79 (60.3)	186 (64.1)	0.451	1.17 (0.77-1.80)
Illiterate	78 (59.5)	138 (47.6)	0.023	1.62 (1.07-2.46)
Unemployed	100 (76.3)	178 (61.4)	0.003	2.03 (1.27-3.24)
Married	103 (78.6)	236 (81.4)	0.509	0.842 (0.51-1.40)
Smoking	38 (29.0)	50 (17.2)	0.006	1.96 (1.20-3.18)
Alcohol	38 (29.0)	59 (20.3)	0.051	1.60 (0.99-2.56)
Physical inactivity	88 (67.2)	143 (49.3)	0.001	2.10 (1.37-3.24)
Blurring of vision	84 (64.1)	131 (45.2)	<0.001	2.17 (1.42-3.32)
Oral hypoglycemic drugs	115 (87.8)	261 (90.0)	0.496	1.25 (0.662-40)
Non-compliant to drugs	112 (85.5)	247 (85.2)	0.931	1.03 (0.57-1.84)
Hyperglycemia (>200mg/dl)	120 (91.6)	227 (78.3)	0.001	3.03 (1.54-5.96)
BMI-Obese>25kg/m2	102 (77.9)	194 (66.9)	0.023	1.74 (1.08-2.81)
High WHR	123 (93.9)	272 (93.8)	0.969	1.02 (0.43-2.40)
Duration of DM >5 years	85 (64.89)	105 (36.2)	<0.001	3.26 (2.12-5.01)

Among the participants with DPN, the mean of age, duration of diabetes, duration of foot symptoms were 60.55 ± 10.55 (years), 9.22 ± 6.91 (years), 6.90 ± 5.94 (years), respectively. As shown in Table 2, the median duration of diabetes, duration of foot symptoms, RBS, BMI and WHR were significantly higher among those with DPN.

**Table 2:** Quantitative factors associated with the risk of diabetic peripheral neuropathy (n=421) *Students t-test; $Mann-Whitney U test; RBS: Random blood sugar; BMI: Body mass index; WHR: Waist-hip ratio; DPN: Diabetic Peripheral Neuropathy; SD: Standard Deviation; IQR: Inter-Quartile Range

Variables	DPN Present (Mean ± SD)/ Median(IQR)	DPN Absent (Mean ± SD) / Median(IQR)	p-value
Age (years)	60.55 ± 10.56	56.72 ± 10.43	0.001*
Duration of Diabetes (years)	8.00 (9.00)	4.00 (6.00)	<0.001^$^
Duration of neurological symptoms (years)	5.00 (6.00)	4.00 (4.00)	<0.001^$^
RBS (mg/dL)	260.00 (42.00)	250.00 (75.00)	0.009^$^
BMI (kg/sq.m)	26.56 (2.17)	26.02 (3.37)	0.119^$^
WHR	0.95 (0.02)	0.95 (0.03)	0.018^$^

In table 3 the variables found to be significantly associated with DPN (p<0.05) from table 1 and table 2 were subjected to multivariate binomial logistic regression analysis. The factors that were significantly associated with DPN were smoking (AOR 3.14; 95% CI 1.73-5.69), mean duration of diabetes more than 5years (AOR 2.74; 95% CI 1.71-4.40); hyperglycemic status (>200mg/dl) (AOR 2.24; 95% CI 1.08-4.64) and unemployment (AOR 2.05; 95% CI 1.11-3.76). 

**Table 3:** Binary logistic regression analysis to determine the predictors of diabetic peripheral neuropathy BMI: body mass index, aOR: Adjusted odds ratio, CI: Confidence interval, B: Unstandardized regression weight, S.E: Standard error

Variable	B	S.E	Wald	p-value	aOR (95%CI)
Age >60 years	0.292	0.257	1.289	0.256	1.339 (0.809-2.217)
Illiteracy	0.275	0.243	1.279	0.258	1.317 (0.817-2.121)
Unemployed	0.715	0.310	5.312	0.021	2.045 (1.113-3.758)
Smoking	1.143	0.304	14.147	<0.001	3.137 (1.729-5.691)
Presence of blurred vision	0.230	0.256	0.809	0.369	1.258 (0.763-2.077)
Duration of Diabetes > 5years	1.007	0.241	17.396	<0.001	2.738 (1.705-4.395)
Hyperglycemia >200 mg/dl	0.805	0.373	4.674	0.031	2.238 (1.078-4.644)
BMI >25kg/m2	0.452	0.267	2.860	0.091	1.571 (0.931-2.651)
Constant	-1.451	0.359	16.373	<0.001	0.234

## Discussion

A facility-based study was conducted among 421 adult DM in a rural health training centre of a medical college in Puducherry using a pre-tested semi-structured questionnaire. The prevalence of DPN determined using MNSI was 31.1%. Bansal et al. at Chandigarh in 2014 has shown the prevalence of DPN to be 29% [13]. A study by D'Souza et al. at Mangalore, Karnataka, in 2014, shows the prevalence of DPN to be 32.2% [14]. A study by Begum et al. [5] in rural Puducherry in 2017 has revealed the prevalence of DPN as 52.9%. A study by Vibha et al. in rural areas of Udupi has reported it as 51.8% [15]. The prevalence of DPN in the present study is in accordance with the studies conducted in urban areas. This could be due to the small geographical region of the Union Territory where rural diabetic people have almost equal access to healthcare facilities as the urban diabetic people. The prevalence of DPN varies widely across various states in India from 15-60% [5]. This difference could be due to the use of different screening instruments to determine the proportion of individuals at risk of developing DPN.

The present study has shown a significant association between advancing age, illiteracy, longer duration of diabetes, unemployment, physical inactivity, smoking, blurred vision, obesity, hyperglycemia and DPN. A study by Vibha et al. in Udupi in 2015 had shown significant association with advancing age, low socio-economic status, sedentary physical activity, longer duration of DM and DPN [15]. A study by Begum et al. [5] done at Puducherry in 2017 had shown significant association with advancing age, smoking and longer duration of the disease, similar to the present study.

Smoking was found to be an independent modifiable risk factor associated with DPN among DM patients in the present study. A study by Mohammad Zubir et al. at Aligarh in 2011 [16] and D'Souza et al. in Karnataka in 2014 [14] had shown a significant association with smoking which was congruent with the present study. Smoking causes oxidative stress and causes damage to the small vessels, which plays a major role in the pathogenesis of DPN [17].

According to the present study, a study by Battula et al. [18] in 2017 at Kurnool had shown a significant association between obesity and DPN. Obesity causes insulin resistance which promotes low-grade inflammation. This inflammation influences endothelial dysfunction and micro-vascular complications [19]. The same patho-physiology holds for a sedentary life-style which increases the likelihood of obesity. A study by Bhansal et al. in Chandigarh in 2014 had shown that other vascular complications like diabetic retinopathy were more in patients with DPN [13]. The present study has shown that patients with DPN have other complications like blurring of vision. DM patients with DPN suffered from a blurring of vision 2.17 times more often than DM patients without DPN. Other micro-vascular complications go hand in hand with DPN.

Studies conducted by Bhansal et al. [13] and Young et al. [20] had shown significant association with advancing age, duration of diabetes and glycemic status. Longer duration of diabetes and poor glycemic control causes accumulation of glycosylation end products, oxidative stress and endothelial damage, all of which have a significant role in the patho-physiology of DPN [21]. In the present study, age more than 60 years, duration of diabetes more than five years, and poor glycemic control were significant associations with DPN. Similarly, studies done by Vibha et al. [15], Begum et al. [5], and Fei Mao et al. [22] had shown age as an independent non-modifiable risk factor associated with DPN. The three main alterations that are involved in the pathogenesis of DPN are inflammation, oxidative stress and mitochondrial dysfunction [23]. All these three alterations are related to the process of ageing [24].

Illiteracy had shown significant association with the development of DPN in the present study. Illiterates tend to have poor self-care in diabetes [25], and also, they are more likely to come under low socio-economic status. All these may be the predisposing factors for illiterates to neglect their physical health, contributing to poor compliance with medications, which in turn causes poor glycemic control.

The previous trial by diabetic complications and the research group showed that early intensive insulin therapy reduced neuropathy symptoms compared to conventional treatment [26]. A previous study by Alvarsson M et al. had shown that early initiation of insulin therapy prolongs endogenous insulin secretion and provides better metabolic control [27]. Nuclear factor-kappa B is the key transcription factor responsible for the transcription of pro-inflammatory cytokines, adhesion molecules and enzymes responsible for producing reactive oxygen species [28]. Insulin infusion significantly suppressed the generation of reactive oxygen species. It decreased concentrations of plasma soluble intercellular adhesion molecule-1 (sICAM-1), monocyte chemo-attractant protein-1 (MCP-1), and plasminogen activator inhibitor-1 (PAI-1), among other observed anti-inflammatory actions [29]. A more timely and selective introduction of insulin replacement therapy could facilitate the achievement and maintenance of euglycemia and reduce disease-associated complications [30].

This study's limitations are that the temporal association could not be assessed due to cross-sectional study design in nature, and recall bias and social desirability could have been there. Some of the variables had large standard deviation indicating a wide variability in the data. There was a lack of nerve conduction studies in the peripheral health care setting, which are the standard gold technique for confirmation of DPN. 

## Conclusions

The present study finds that one in three diabetic patients are at risk of diabetic peripheral neuropathy. Smoking, physical inactivity, longer duration of diabetes and uncontrolled hyperglycemia were the significant independent predictors associated with DPN. The policymakers can make the screening for DPN an integral part of the services offered in every non-communicable disease clinic and consider these risk factors for awareness generation for a target population.
